# White matter microstructure and its relation to clinical features of obsessive–compulsive disorder: findings from the ENIGMA OCD Working Group

**DOI:** 10.1038/s41398-021-01276-z

**Published:** 2021-03-17

**Authors:** Fabrizio Piras, Federica Piras, Yoshinari Abe, Sri Mahavir Agarwal, Alan Anticevic, Stephanie Ameis, Paul Arnold, Nerisa Banaj, Núria Bargalló, Marcelo C. Batistuzzo, Francesco Benedetti, Jan-Carl Beucke, Premika S. W. Boedhoe, Irene Bollettini, Silvia Brem, Anna Calvo, Kang Ik Kevin Cho, Valentina Ciullo, Sara Dallaspezia, Erin Dickie, Benjamin Adam Ely, Siyan Fan, Jean-Paul Fouche, Patricia Gruner, Deniz A. Gürsel, Tobias Hauser, Yoshiyuki Hirano, Marcelo Q. Hoexter, Mariangela Iorio, Anthony James, Y. C. Janardhan Reddy, Christian Kaufmann, Kathrin Koch, Peter Kochunov, Jun Soo Kwon, Luisa Lazaro, Christine Lochner, Rachel Marsh, Akiko Nakagawa, Takashi Nakamae, Janardhanan C. Narayanaswamy, Yuki Sakai, Eiji Shimizu, Daniela Simon, Helen Blair Simpson, Noam Soreni, Philipp Stämpfli, Emily R. Stern, Philip Szeszko, Jumpei Takahashi, Ganesan Venkatasubramanian, Zhen Wang, Je-Yeon Yun, ENIGMA OCD Working Group, Francesca Assogna, Rosa Calvo, Stella J. de Wit, Morgan Hough, Masaru Kuno, Euripedes C. Miguel, Astrid Morer, Christopher Pittenger, Sara Poletti, Enrico Smeraldi, João R. Sato, Aki Tsuchiyagaito, Susanne Walitza, Ysbrand D. van der Werf, Daniela Vecchio, Mojtaba Zarei, Dan J. Stein, Neda Jahanshad, Paul M. Thompson, Odile A. van den Heuvel, Gianfranco Spalletta

**Affiliations:** 1 Laboratory of Neuropsychiatry, Department of Clinical and Behavioral Neurology, IRCCS Santa Lucia Foundation, Rome, Italy; 2 Department of Psychiatry, Graduate School of Medical Science, Kyoto Prefectural University of Medicine, Kyoto, Japan; 3 Obsessive-Compulsive Disorder (OCD) Clinic, Department of Psychiatry, National Institute of Mental Health & Neurosciences, Bangalore, India; 4 Department of Psychiatry, Yale University School of Medicine, New Haven, CT USA; 5 Child, Youth and Emerging Adult Program, Campbell Family Mental Health Research Institute, Centre for Addiction and Mental Health (CAMH), Toronto, ON Canada; 6 Department of Psychiatry, Hospital for Sick Children, Toronto, ON Canada; 7 Department of Psychiatry, University of Toronto, Toronto, ON Canada; 8 Mathison Centre for Mental Health Research & Education, Hotchkiss Brain Institute, Cumming School of Medicine, University of Calgary, Calgary, Alberta Canada; 9 Magnetic Resonance Image Core Facility, Institut d’Investigacions Biomèdiques August Pi i Sunyer (IDIBAPS), Barcelona, Spain; 10 Centre de Diagnostic per la Imatge (CDIC), Hospital Clínic de Barcelona, Barcelona, Spain; 11 Instituto e Departamento de Psiquiatria, Hospital das Clinicas HCFMUSP, Faculdade de Medicina, Universidade de Sao Paulo, Sao Paulo, SP Brazil; 12 Psychiatry and Clinical Psychobiology, Division of Neuroscience, Scientific Institute Ospedale San Raffaele, Milano, Italy; 13 Department of Psychology, Humboldt-Universität zu Berlin, Berlin, Germany; 14 K8 Department of Clinical Neuroscience, Karolinska Institutet, Stockholm, Sweden; 15 Amsterdam University Medical Centers, Vrije Universiteit, Department of Psychiatry, Amsterdam Neuroscience, Amsterdam, The Netherlands; 16 Amsterdam university medical centers, Vrije Universiteit, Department of Anatomy & Neurosciences, Amsterdam Neuroscience, Amsterdam, The Netherlands; 17 Department of Child and Adolescent Psychiatry and Psychotherapy, Psychiatric Hospital, University of Zurich, Zurich, Switzerland; 18 Institute of Human Behavioral Medicine, SNU-MRC Seoul, Republic of Korea; 19 Campbell Family Mental Health Research Institute, Centre for Addiction and Mental Health (CAMH), Toronto, ON Canada; 20 Department of Neuroscience and Graduate School, Icahn School of Medicine at Mount Sinai, New York, NY USA; 21 SAMRC Unit on Anxiety & Stress Disorders, Department of Psychiatry and Neuroscience Institute, University of Cape Town, Cape Town, South Africa; 22 Department of Neuroradiology, Klinikum rechts der Isar, Technische Universität München, München, Germany; 23 Research Center for Child Mental Development, Chiba University, Chiba, Japan; 24 Center of Excellence on Aging and Translational Medicine - CeSI-MeT, Chieti, Italy; 25 Department of Psychiatry, Oxford University, Oxford, UK; 26 Department of Psychology, Freie Universität zu Berlin, Berlin, Germany; 27 Maryland Psychiatric Research Center, Department of Psychiatry, University of Maryland School of Medicine, Baltimore, MD USA; 28 Department of Child and Adolescent Psychiatry and Psychology, Institute of Neurosciences, Hospital Clínic Universitari, Barcelona, Spain; 29 Institut d’Investigacions Biomèdiques August Pi i Sunyer (IDIBAPS), Barcelona, Spain; 30 Columbia University Irving Medical Center, Columbia University, New York, NY USA; 31 The new York State Psychiatric Institute, New York, NY USA; 32 Pediatric OCD Consultation Clinic, Anxiety Treatment and Research Center, McMaster University, Hamilton, Ontario Canada; 33 MR-Center of the Department of Psychiatry, Psychotherapy and Psychosomatics and the Department of Child and Adolescent Psychiatry, Psychiatric Hospital of the University of Zurich, Zurich, Switzerland; 34 Department of Psychiatry, Icahn School of Medicine at Mount Sinai, New York, NY USA; 35 Shanghai Mental Health Center, Shanghai Jiao Tong University School of Medicine, Shanghai, PR China; 36 Imaging Genetics Center, Mark and Mary Stevens Neuroimaging & Informatics Institute, Keck School of Medicine of the University of Southern California, Marina del Rey, USA; 37 Division of Neuropsychiatry, Menninger Department of Psychiatry and Behavioral Sciences, Baylor College of Medicine, Houston, TX USA; 38 CIBERSAM, Barcelona, Spain; 39 Highfield Unit, Warneford Hospital, Oxford, UK; 40 Center of Mathematics, Computation, and Cognition, Universidade Federal do ABC, Santo André, São Paulo Brazil; 41 Institute of Medical Science and Technology, Shahid Beheshti University, Tehran, Iran

**Keywords:** Neuroscience, Psychiatric disorders

## Abstract

Microstructural alterations in cortico-subcortical connections are thought to be present in obsessive–compulsive disorder (OCD). However, prior studies have yielded inconsistent findings, perhaps because small sample sizes provided insufficient power to detect subtle abnormalities. Here we investigated microstructural white matter alterations and their relation to clinical features in the largest dataset of adult and pediatric OCD to date. We analyzed diffusion tensor imaging metrics from 700 adult patients and 645 adult controls, as well as 174 pediatric patients and 144 pediatric controls across 19 sites participating in the ENIGMA OCD Working Group, in a cross-sectional case-control magnetic resonance study. We extracted measures of fractional anisotropy (FA) as main outcome, and mean diffusivity, radial diffusivity, and axial diffusivity as secondary outcomes for 25 white matter regions. We meta-analyzed patient-control group differences (Cohen’s *d*) across sites, after adjusting for age and sex, and investigated associations with clinical characteristics. Adult OCD patients showed significant FA reduction in the sagittal stratum (*d* = −0.21, *z* = −3.21, *p* = 0.001) and posterior thalamic radiation (*d* = −0.26, *z* = −4.57, *p* < 0.0001). In the sagittal stratum, lower FA was associated with a younger age of onset (*z* = 2.71, *p* = 0.006), longer duration of illness (*z* = −2.086, *p* = 0.036), and a higher percentage of medicated patients in the cohorts studied (*z* = −1.98, *p* = 0.047). No significant association with symptom severity was found. Pediatric OCD patients did not show any detectable microstructural abnormalities compared to controls. Our findings of microstructural alterations in projection and association fibers to posterior brain regions in OCD are consistent with models emphasizing deficits in connectivity as an important feature of this disorder.

## Introduction

Abnormalities in cerebral white matter (WM) are relevant to models of anomalous brain circuitry that posit deficits in connectivity in obsessive–compulsive disorder (OCD). OCD has a childhood onset in over 50% of all cases, and most childhood-onset OCD cases persist into adulthood^1^. Diffusion tensor imaging (DTI) allows the study of WM at the microstructural level through the analysis of intrinsic, three-dimensional diffusion properties of water within brain tissues^2^. Prior DTI studies in OCD^3–5^ suggest that microstructural alterations are present in a number of WM areas. However, results across studies are inconsistent, with contrasting or conflicting effects of OCD on DTI metrics^6^. Sources of heterogeneity may include methodological factors (e.g., imaging acquisition and data processing), clinical characteristics, and variations in demographic or socioeconomic factors. More importantly, sample size variations may impact reported findings, as small studies may have insufficient power to detect subtle alterations^7^.

Brain imaging consortia offer new opportunities, pooling data and findings from around the world to achieve an appropriate sample size. The OCD working group of the Enhancing Neuro-Imaging Genetics through Meta-Analysis (ENIGMA) consortium^8^, is one such collaboration. Previous findings from the working group focused on subcortical and cortical brain gray matter abnormalities, using subcortical volumes, cortical thickness, and surface area quantification algorithms. An initial analysis of data from 3589 individuals showed distinct subcortical volume abnormalities in adults (smaller hippocampal and larger pallidal volumes) and unmedicated children (larger thalamic volume) with OCD^9^. The second study focused on cortical gray matter differences and showed a lower surface area for the transverse temporal cortex and a thinner inferior parietal cortex in adult patients. In pediatric OCD patients compared to healthy controls, significantly thinner inferior and superior parietal cortices were found^10^. Medication status was associated with structural differences in both pediatric and adult OCD.

Here we aimed to investigate WM microstructural alterations in adult and pediatric OCD using data from the ENIGMA OCD working group, in the subset of participants that had collected diffusion magnetic resonance imaging (MRI). DTI metrics in 700 adult patients were compared to those of 645 adult controls, and separately, 174 pediatric patients were compared to 144 pediatric controls. Analyses also aimed to investigate associations between WM microstructure and demographic and clinical variables. As prior meta-analytic findings in frontal and callosal regions have been inconsistent (with either higher^11^ or lower^3,11^ fractional anisotropy (FA) in anterior midline tracts), with more homogenous findings for fronto-temporal and fronto-parietal intra-hemispheric bundles, we expected to find microstructural alterations (as reflected by lower FA^3,4,6,11^) in the long tracts connecting frontal regions to posterior temporal, parietal and occipital association cortices.

## Methods

### Study dataset

The ENIGMA OCD Working Group includes 19 international research institutes. Previous literature (including studies from the present Working Group^9,10^) showed different patterns of effects in pediatric and adult cohorts; thus, we performed separate meta-analyses for adult and pediatric data. Globally, we analyzed data from 1345 adults (including 700 OCD patients and 645 controls, aged ≥18) and 318 children (including 174 OCD patients and 144 controls). The diagnosis of psychiatric disorders including OCD and other comorbid conditions (if any) was made using a structured or semi-structured interview; the Structured Clinical Interview for DSM-IV [(First et al.^12^) *n* = 9 datasets], the Mini-International Neuropsychiatric Interview [(Sheehan et al.^13^) *n* = 3 datasets], or the schedule for affective disorders and schizophrenia for school-aged children: Present and Lifetime Version [K-SADS-PL^14^; *n* = 7 datasets]. Patients were administered the Yale-Brown Obsessive–Compulsive Scale (YBOCS)^15^ and the Child YBOCS^16^ to assess symptom severity. These tools are clinician-rated, 10-item scales, with each item rated from 0 (no symptoms) to 4 (extreme symptoms; total range, 0–40), with separate subtotals for the severity of obsessions and compulsions.

Common exclusion criteria across sites included: (1) history of psychoactive substance dependence or abuse during lifetime, (2) history of neurologic illness or brain injury, (3) presence of any brain pathology as instantiated by standard magnetic resonance imaging (MRI) exams (including T1-weighted and standard clinical sequences), (4) dementia diagnosis according to DSM-IV-TR criteria.

Tables 1 and 2 show the demographic and clinical characteristics of the participants from each site.

**Table 1:** Demographic and clinical characteristics of patients with obsessive–compulsive disorder (OCD) and control subjects.

Characteristics	Adult OCD sample (*n* = 700)	Adult HC sample (*n* = 645)	Pediatric OCD sample (*n* = 174)	Pediatric HC sample (*n* = 144)
Age (years)	31.4 ± 9.9	30.7 ± 10	14.5 ± 2.3	14.3 ± 2.5
OCD illness severity score	25 ± 7.1	–	20.7 ± 7.8	–
Age at onset	19.1 ± 8.4	–	13.1 ± 5.3	–
	*N* (%)	*N* (%)	*N* (%)	*N* (%)
Male	405 (58)	378 (59)	94 (53)	74 (51.3)
Medication use at time of scan	269 (39)	–	112 (64)	–
Current comorbid disorders				
Anxiety	74^a^ (11)	–	27^b^ (23)	–
Major depression	60^c^ (10)	–	10^b^ (9)	–
OCD symptom dimension				
Aggressive/checking	418^d^ (79)	–	82^e^ (75)	–
Contamination/cleaning	358^d^ (68)	–	74^e^ (68)	–
Symmetry/ordering	378^d^ (62)	–	72^e^ (66)	–
Sexual/religious	229^d^ (44)	–	47^e^ (43)	
Hoarding	115^d^ (22)	–	45^e^ (41)	–

^a^Data available for 625 patients.

^b^Data available for 117 patients.

^c^Data available for 610 patients.

^d^Data available for 525 patients.

^e^Data available for 109 patients.

**Table 2:** (a) Breakdown, by site, of clinical characteristics of adult patients with obsessive–compulsive disorder (OCD) in the ENIGMA OCD Working Group samples; (b) Breakdown, by site, of clinical characteristics of pediatric patients with obsessive–compulsive disorder (OCD) in the ENIGMA OCD Working Group samples.

Site	OCD/HC (N)	Medicated (%)	Age of onset	Duration of illness	YBOCS score	Lifetime anxiety (%)	Lifetime depression %
(a)							
Amsterdam	38/34	0	15.1 ± 6.8	23.7 ± 12.8	21.3 ± 6.1	42.1	47.4
Bangalore	158/131	39.9	22.3 ± 7.7	7.2 ± 5.2	25.5 ± 6.5	8.9	7
Capetown	22/23	40.9	13.2 ± 5.7	17.2 ± 11.5	23 ± 4.2	0	0
Kyoto	35/41	0	25.2 ± 9	7.7 ± 6.2	21.9 ± 6.6	8.6	0
Milan	63/65	60.3	15.6 ± 6.2	18.9 ± 11.6	31.4 ± 5.2	1.6	7.9
Mount Sinai	16/18	81.3	12.4 ± 5.7	15.1 ± 6.7	19.9 ± 5.9	50	18.8
Munich	73/60	60.3	17.6 ± 6.7	13.5 ± 10.3	20.8 ± 6.2	8.2	23.3
Rome	77/111	94.8	16.8 ± 8	17.3 ± 12.8	23.2 ± 9.3	10.4	9.1
Sao Paulo	37/30	43.2	12.8 ± 5.9	26.3 ± 13.5	29.2 ± 6.2	73	83.8
Shangai	83/45	0	24 ± 9.9	6 ± 5.9	26.2 ± 4.7	0	0
Seoul	98/87	13.3	19.1 ± 7.2	6.2 ± 7	25.8 ± 6.9	1	2

(b)							
Bangalore	13/13	85	12.8 ± 1.9	1.5 ± 1	21 ± 7.6	23.1	23.1
Barcelona	52/27	78.8	12.2 ± 2.5	1.5 ± 2.1	20.8 ± 7.6	28.8	5.8
British Columbia	13/16	86.7	11.1 ± 3.5	3.3 ± 2.7	14 ± 6	33.3	0
Calgary	19/18	0	NA	NA	23.1 ± 4.7	NA	NA
Chiba	20/6	40	11.9 ± 2.4	2.1 ± 1.8	27 ± 6.2	10	0
Oxford	13/18	63.6	11.7 ± 3	4.7 ± 3.2	20 ± 7.3	31.8	22.7
Yale	22/23	52.2	NA	NA	26.9 ± 4.5	43.5	39.1
Zurich	22/23	57.1	11.1 ± 2.4	4.7 ± 2.3	16.1 ± 10.1	42.9	0

*YBOCS* Yale-Brown Obsessive–Compulsive Scale, *NA* not available.

All local IRBs approved the use of measures extracted from completely anonymized data.

### Image acquisition and processing

Harmonized preprocessing, including brain extraction, eddy current correction, movement correction, echo-planar imaging-induced distortion correction and tensor fitting, was carried out at each site, using protocols and quality control pipelines provided by the ENIGMA-DTI working group (http://enigma.ini.usc.edu/protocols/dti-protocols/) and already employed to pool harmonized DTI analyses from around the world^17–19^.

Once tensors were estimated, each site conducted a harmonized image analysis for FA quantification using the ENIGMA-DTI protocol, consisting of the tract-based spatial statistics (TBSS)^20^ analytic method modified to project individual FA values to the ENIGMA-DTI skeleton. Tract-wise regions of interest (ROIs), derived from the Johns Hopkins University^21^ WM parcellation atlas, were used to extract the mean FA across the full skeleton and mean FA values for 25 ROIs.

Diffusivity measures (i.e., mean diffusivity (MD), axial diffusivity (AD), and radial diffusivity (RD)) were also derived for secondary analysis (i.e., the analyses were performed only in those WM regions, if any, where FA was significant). In the main analyses, we combined left and right ROI across hemispheres, as we had no a priori hypotheses regarding lateralized effects on FA.

### Statistical analysis

At each site, Cohen’s *d* effect sizes were calculated for differences in FA between patients and healthy controls. Age, sex, age-by-sex interaction, and quadratic covariates of age^2^ and age^2^-by-sex interaction were included in the model, as linear and nonlinear age and sex interactions have been reported for FA^17^. Subsequently, a random effects meta-analysis was run at the coordinating site using Comprehensive Meta-Analysis (CMA, version 2, Biostat, Englewood, NJ) to combine individual site effect sizes. Heterogeneity scores (*I*^2^; lower values indicate lower variance in the effect size estimates across studies) were also computed for each test.

Effect sizes are reported as overall Cohen’s *d* values for case/control effects and *z*-scores, and were considered significant if *p* < 0.05. The stability of the overall effect size estimate was tested using a ‘leave one out’ sensitivity analysis. This analysis shows how the overall effect size changes if one dataset at a time is removed, assessing whether potential results are site-dependent with between-sites variations potentially deriving from variability in study population characteristics (sampling error). Furthermore, to ascertain whether the estimated effect size varied as a function of clinical characteristics, mixed-effects meta-regressions were performed on FA, using age of onset, duration of illness, symptom severity, and percentage of medicated patients in the patients’ dataset as regressors. The influence of medication status was also explored through a mixed-effects sub-group analysis, comparing effect sizes in medicated (*n* = 8) and unmedicated (*n* = 3) patient cohorts. These analyses were primarily run in those WM areas where effect sizes were significant and stable according to the leave-one-out analyses. Ancillary analyses explored the effect of clinical variables on FA also in WM areas where only partly stable results were observed (i.e., where the removal of 1 or 2 studies affected significance)

## Results

Demographics and clinical characteristics of the participants in each site are shown in Tables 1 and 2.

### Adult Cohort

**Fig. 1:**
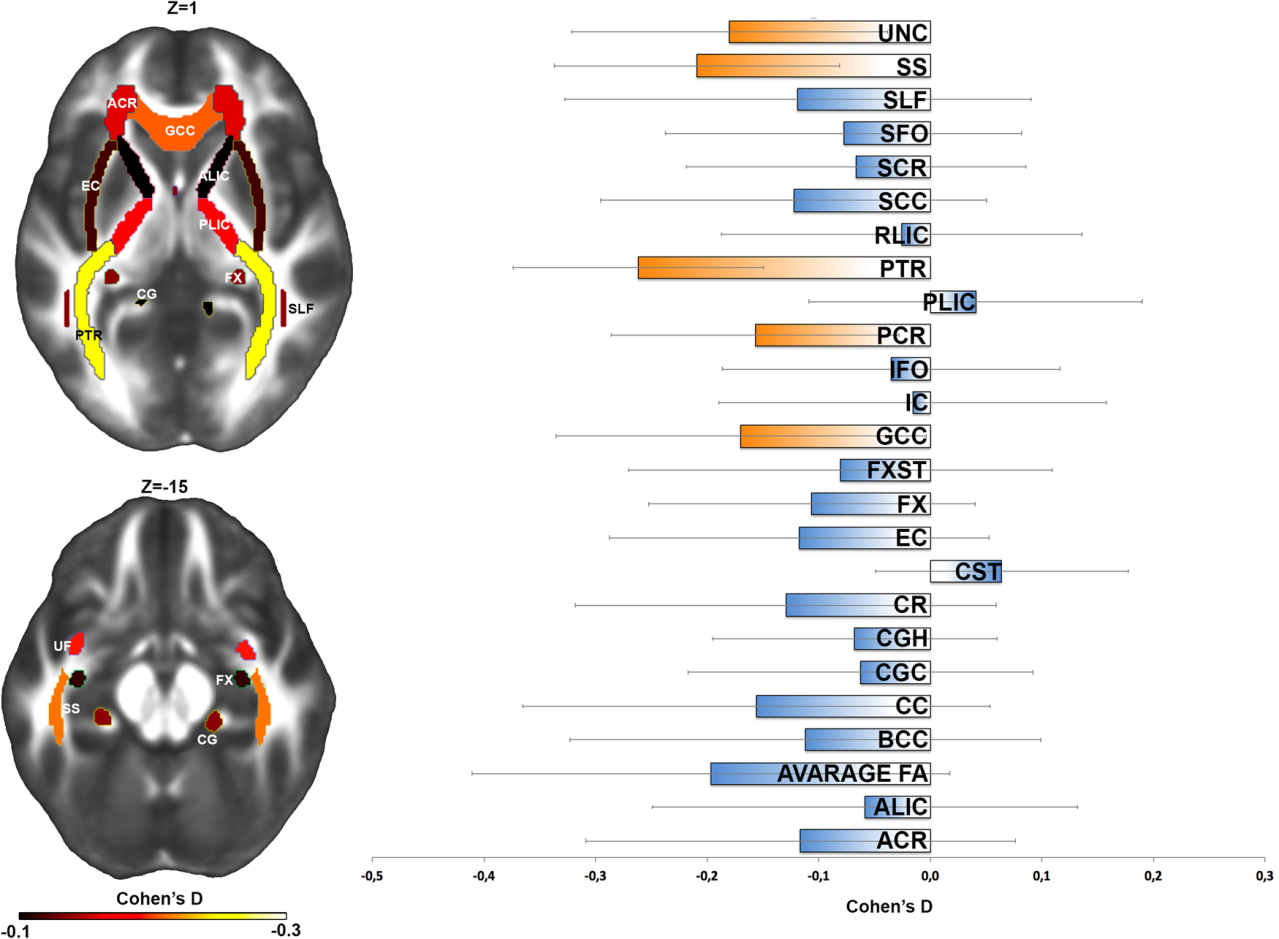
Left panel—fractional anisotropy (FA) differences between OCD patients and healthy controls for 25 white matter (WM) regions. Gradient bar indicates Cohen’s *d* effect sizes after meta-analysis. Right Panel- Cohen’s *d* effect sizes after meta-analysis, including age, sex, age × sex, age^2^, and age^2^ × sex as covariates. Error bars represent 95% confidence intervals. Significant regions are highlighted in orange.

Table 3 indicates the 5 out of 25 regions with lower FA in patients compared to controls. These are the genu of the corpus callosum (GCC, *d* = −0.17, *z* = −2, *p* = 0.045), the posterior corona radiata (PCR, *d* = −0.16, *z* = −2.38, *p* = 0.017), the posterior thalamic radiation (PTR, *d* = −0.261, *z* = −4.57, *p* < 0.0001), the sagittal stratum (SS, *d* = −0.21, *z* = −3.21, *p* = 0.001) and the uncinate fasciculus (UNC, *d* = −0.18, *z* = −2.49, *p* = 0.013) (see Fig. 1).

**Table 3:** FA meta-analysis metrics for the adult sample.

	Effect size and 95% confidence interval						Heterogeneity		
ROI	Cohen’s *d*	S.E.	Lower limit	Upper limit	*z* value	*p* value	*q* value	*p* value	*I* squared
ACR	−0.1164	0.0983	−0.3091	0.0763	−1.1839	0.2364	29.1988	0.0012	65.7520
ALIC	−0.0584	0.0971	−0.2488	0.1320	−0.6013	0.5477	28.5200	0.0015	64.9369
AverageFA	−0.1968	0.1091	−0.4107	0.0171	−1.8036	0.0713	35.9099	0.0001	72.1525
BCC	−0.1119	0.1076	−0.3227	0.0990	−1.0398	0.2984	34.9932	0.0001	71.4230
CC	−0.1558	0.1067	−0.3650	0.0533	−1.4606	0.1441	34.4018	0.0002	70.9317
CGC	−0.0626	0.0789	−0.2173	0.0920	−0.7938	0.4273	18.9453	0.0410	47.2164
CGH	−0.0677	0.0650	−0.1951	0.0598	−1.0404	0.2982	13.2641	0.2093	24.6083
CR	−0.1294	0.0962	−0.3179	0.0591	−1.3454	0.1785	27.9259	0.0019	64.1910
CST	0.0641	0.0577	−0.0490	0.1772	1.1106	0.2667	10.8843	0.3666	8.1241
EC	−0.1173	0.0868	−0.2873	0.0528	−1.3513	0.1766	22.7693	0.0116	56.0812
FX	−0.1063	0.0745	−0.2523	0.0398	−1.4259	0.1539	16.9566	0.0753	41.0259
FXST	−0.0804	0.0968	−0.2701	0.1093	−0.8307	0.4062	28.3405	0.0016	64.7148
GCC	−0.1696	0.0845	−0.3352	−0.0041	−2.0085	0.0446	21.5584	0.0175	53.6144
IC	−0.0158	0.0887	−0.1896	0.1581	−0.1776	0.8590	23.8155	0.0081	58.0106
IFO	−0.0350	0.0772	−0.1864	0.1164	−0.4531	0.6505	18.1854	0.0519	45.0108
PCR	−0.1570	0.0660	−0.2863	−0.0277	−2.3803	0.0173	13.5590	0.1941	26.2484
PLIC	0.0406	0.0763	−0.1090	0.1903	0.5321	0.5947	17.7645	0.0591	43.7078
PTR	−0.2619	0.0573	−0.3742	−0.1495	−4.5689	0.0000	10.7084	0.3807	6.6151
RLIC	−0.0256	0.0823	−0.1869	0.1356	−0.3117	0.7553	20.5308	0.0246	51.2926
SCC	−0.1223	0.0882	−0.2952	0.0506	−1.3868	0.1655	23.5299	0.0090	57.5008
SCR	−0.0664	0.0776	−0.2184	0.0856	−0.8565	0.3917	18.3052	0.0500	45.3707
SFO	−0.0776	0.0813	−0.2370	0.0818	−0.9545	0.3399	20.0600	0.0287	50.1495
SLF	−0.1189	0.1066	−0.3278	0.0901	−1.1149	0.2649	34.3842	0.0002	70.9169
SS	−0.2090	0.0651	−0.3367	−0.0814	−3.2100	0.0013	13.2328	0.2109	24.4304
UNC	−0.1799	0.0723	−0.3216	−0.0382	−2.4885	0.0128	15.9772	0.1003	37.4109

Cohen’s *d* values, their s.e., lower and upper limits, *p* values and *I*^2^ (heterogeneity) values after meta-analysis for differences between OCD patients and healthy controls.

Heterogeneity scores revealed a significant high variance for GCC (*I*^2^ = 53.61, *p* = 0.018) only, whereas PCR (*I*^2^ = 26.25, *p* = 0.19), PTR (*I*^2^ = 6.61, *p* = 0.38), SS (*I*^2^ = 24.43, *p* = 0.21), and UNC (*I*^2^ = 37.4, *p* = 0.1) showed non-significant variance between sites.

The sensitivity analysis showed that PTR and SS were the only WM tracts where the removal of individual datasets did not affect significance. For the other WM tracts results are more controversial, since for GCC the exclusion of six sites determined a loss of significance of the model, while for PCR and UNC the exclusion of two sites determined a loss of significance of the model (see Supplementary Table 1).

As secondary analyses, we also investigated diffusivity measures (i.e., MD, AD, and RD) in those WM regions where FA was significantly reduced in OCD. Results revealed that patients diagnosed with OCD showed higher MD in the SS (*d* = 0.21, *z* = 2.75, *p* = 0.006) and higher RD in PTR and SS (*d* = 0.16, *p* = 0.002 for PTR and *d* = 0.21, *p* = 0.007 for SS). No significant results were found for AD.

**Fig. 2:**
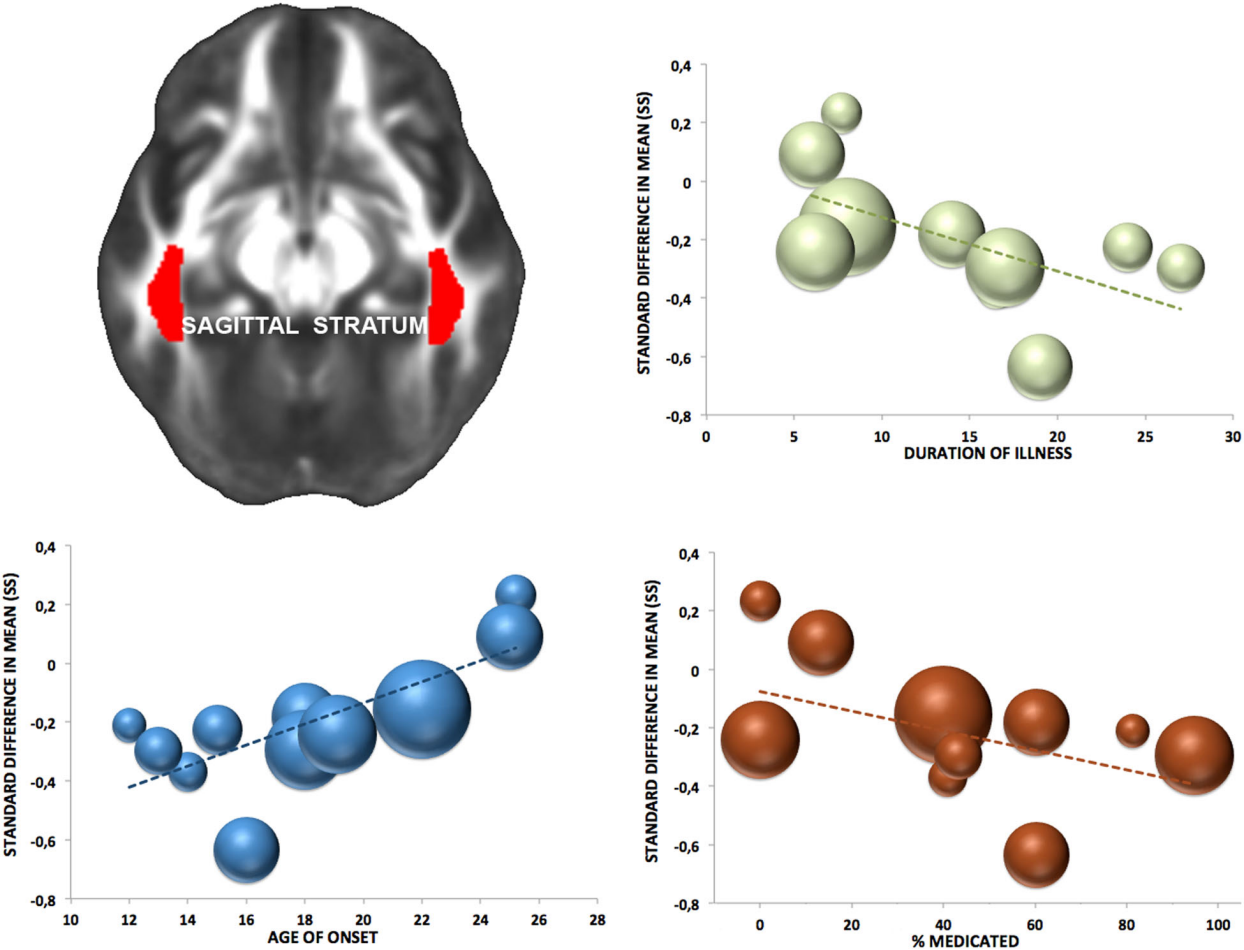
Association between FA reduction (in OCD adult patients) in the sagittal stratum and illness duration (*green dots*), age of onset (*blue dots*) and percentage of medicated patients across the 11 ENIGMA-OCD sites. Sphere magnitude indicates sample size.

As stated, meta-regressions were primarily run in regions where effect sizes were significant and stable (i.e., PTR and SS). In the SS of adults diagnosed with OCD, lower FA was significantly associated with younger age of onset (*z* = 2.71, *p* = 0.006), longer duration of illness (*z* = −2.09, *p* = 0.036) and a higher percentage of medicated patients (*z* = −1.98, *p* = 0.047; see Fig. 2). Mixed-effects sub-groups analysis showed a significant difference (*q* value_(df = 1)_ = 5.27, *p* = 0.022) between the effect sizes in medicated (*N* = 544, *d* = −0.274, *p* = <0.0001) and unmedicated (*N* = 158, *d* = 0.046, *p* = 0.72) patients. No relationship was found between FA values of the PTR and clinical measures. No relationship was found between FA and YBOCS scores.

Ancillary analyses in those WM areas where results were unstable according to the leave-one-out analyses (i.e., PCR and UNC), revealed that lower FA was associated with longer duration of illness (*z* = −2.308, *p* = 0.021) and higher percentage of medicated patients (*z* = −2.817, *p* = 0.005) in the PCR, and with higher percentage of medicated patients (*z* = −2.453, *p* = 0.014) in the UNC.

### Pediatric cohort

In the pediatric cohort, patients showed no detectable FA abnormalities in any of the regions studied (see Table 4 for statistical details).

**Table 4:** FA meta-analysis metrics for the pediatric sample.

	Effect size and 95% confidence interval						Heterogeneity		
ROI	Cohen’s *d*	S.E.	Lower limit	Upper limit	*z* value	*p* value	*q* value	*p* value	*I* squared
ACR	0.035	0.111	−0.183	0.254	0.318	0.751	4.154	0.762	0.000
ALIC	0.103	0.145	−0.180	0.386	0.711	0.477	11.207	0.130	37.538
AverageFA	0.077	0.111	−0.142	0.295	0.687	0.492	5.421	0.609	0.000
BCC	0.011	0.138	−0.260	0.282	0.079	0.937	10.316	0.171	32.143
CC	−0.013	0.117	−0.242	0.217	−0.108	0.914	7.596	0.370	7.844
CGC	0.111	0.112	−0.107	0.330	0.997	0.319	6.212	0.515	0.000
CGH	−0.017	0.143	−0.296	0.263	−0.116	0.908	10.928	0.142	35.946
CR	0.022	0.111	−0.196	0.240	0.197	0.844	5.042	0.655	0.000
CST	−0.110	0.153	−0.409	0.189	−0.723	0.469	12.410	0.088	43.593
EC	−0.025	0.111	−0.244	0.193	−0.226	0.821	5.000	0.660	0.000
FX	−0.119	0.111	−0.337	0.099	−1.072	0.284	1.237	0.990	0.000
FXST	−0.095	0.134	−0.357	0.168	−0.707	0.480	9.725	0.205	28.020
GCC	−0.018	0.111	−0.236	0.201	−0.158	0.875	4.635	0.704	0.000
IC	0.013	0.111	−0.205	0.231	0.116	0.907	3.551	0.830	0.000
IFO	−0.025	0.111	−0.243	0.193	−0.228	0.819	2.937	0.891	0.000
PCR	0.078	0.116	−0.149	0.305	0.676	0.499	7.443	0.384	5.958
PLIC	0.026	0.111	−0.192	0.244	0.237	0.813	2.079	0.955	0.000
PTR	−0.004	0.201	−0.398	0.390	−0.022	0.982	21.204	0.003	66.987
RLIC	−0.067	0.111	−0.285	0.151	−0.602	0.547	5.021	0.657	0.000
SCC	−0.036	0.112	−0.255	0.183	−0.321	0.748	6.867	0.443	0.000
SCR	−0.040	0.111	−0.258	0.178	−0.357	0.721	4.165	0.761	0.000
SFO	0.100	0.155	−0.203	0.404	0.649	0.516	12.757	0.078	45.127
SLF	−0.089	0.116	−0.317	0.139	−0.763	0.446	7.519	0.377	6.904
SS	0.006	0.146	−0.280	0.292	0.041	0.967	11.433	0.121	38.775
UNC	0.053	0.119	−0.181	0.286	0.441	0.659	7.840	0.347	10.711

Cohen’s *d* values, their s.e., lower and upper limits, *p* values and *I*^2^ (heterogeneity) values after meta-analysis for differences between OCD patients and healthy controls.

## Discussion

In the largest coordinated meta-analysis of WM in OCD to date, we demonstrated specific regional WM alterations in adults with OCD, with lower FA in GCC, PCR, PTR, SS, and UNC. Such results were stable and independent of sampling error in the PTR and SS only. Secondary analyses on other diffusion parameters revealed that OCD showed higher MD in the SS and higher RD in PTR and SS.

Meta-regressions indicated that lower FA in the SS is associated with younger age at onset, longer duration of illness, and being on medication, but not with symptom severity suggesting that—as observed for cortical thickness and subcortical volumes in OCD—, the reported alterations may be markers of the disorder. We did not find case-control differences in WM microstructure of pediatric subjects.

A role for cerebral WM and oligodendrocytes (the myelinating cells of the central nervous system) in the pathophysiology of many psychiatric disorders has been supported by growing research evidence^4,22^, suggesting abnormalities of myelination status as a possible pathogenic mechanism^23^. Specifically, altered myelin-related maturational growth may explain the enhanced risk for psychiatric disorders during the transition from childhood to adulthood^24,25^, an age window of intense ongoing brain development^23^.

Although FA is a general measure of microstructure—including variation in regional myelination levels, such as axon demyelination or loss, myelin loss or increased extracellular space—it does not provide a physiologically specific explanation of WM abnormalities^26^. In our study, higher MD and RD (and the absence of changes in AD) in the same bundles supports the hypothesis that lower FA reflects a disruption of myelin sheaths^27,28^, given that RD is a putative myelin marker^26^.

The association between myelin degradation in the SS and longer illness duration together with the absence of a detectable alteration in pediatric patients, suggest that neuroplastic changes may reflect prolonged symptomatology, since compulsively engaging in a particular behavior or cognitive process has been suggested to alter brain structure^29,30^. Moreover, symptoms indicative of obsessive–compulsive traits are related to individual myelination over time, even in otherwise healthy samples. This suggests that the mechanisms underlying compulsivity have long-lasting effects on brain development, possibly affecting myelination trajectories during adolescence, with enduring effects into adulthood^23^. Alternatively, the paucity of extrinsic factors regulating the development of myelinating glia^31^ could have driven the altered myelination and its association with prolonged illness. Indeed, impoverished environment is both the cause and the consequence of mental illness in general, and of compulsivity in particular^32^.

Lower FA in the SS was related to medication status (this was true also for PCR and UNC), and present only in the cohort of medicated patient. Moreover, the effect remained significant across combinations of datasets when only medicated patients were considered. Therefore, we cannot rule out the possibility that medication impacts WM microstructure. Indeed, drug-induced reductions in the FA of several WM tracts may be seen in OCD^33^ and long-term treatment exposure may negatively influence the proliferation of oligodendrocytes and their myelination of axons^27,34–36^. Our findings are consistent with a previous large multimodal meta-analysis^3^ where increased WM volume and decreased FA were especially pronounced in OCD samples with a high proportion of medicated patients. That said, given the cross-sectional nature of the present study, our interpretation here is a tentative one, and requires confirmation with a longitudinal design.

PTR and the SS (but also the PCR) convey projection fibers to the posterior part of the brain. Thus, our results strengthen the hypothesis that OCD involves abnormalities affecting an extensive network of regions^11,37^. Both bundles project to posterior parietal, temporal and occipital cortices, and include many major association fibers (including the UNC) where altered microstructure may be related to clinical phenomenology^38,39^. Our findings are consistent with a range of work indicating altered connectivity outside the fronto-striatal circuit in OCD. For example, results of a multimodal structural imaging study suggested that patients with OCD show significant alterations of the interrelated gray and WM networks over occipital and parietal cortices, frontal interhemispheric connections, and cerebellum^40^. Also, decreased functional connectivity in the occipital cortex, temporal cortex, and cerebellum has been shown in OCD^41^. Finally, there is evidence in OCD of associations between structural and functional alterations in a complex network including, beyond orbitofrontal and cingulate areas, temporal and occipital cortices^42^.

We only partially replicate previous large meta-analytic findings demonstrating the validity of both the classic fronto-striatal model of the disorder^3^ and the more recent multiple brain system approach^11,43^. The present findings are based on the TBSS technique, which by reducing WM tracts into a skeleton, confines statistical testing to a selective group of voxels through a constrained local search for maximal FA. Such an algorithm may therefore produce more consistent results, but also more conservative ones, thus explaining the lack of case/control group differences in more anterior WM structures found in previous meta-analysis where whole-brain WM volumetric and FA studies were combined^3^.

Notably, in our study WM microstructural alterations in OCD were associated with age at scanning. Specifically, WM alterations were observed in the adult cohort only, and were associated with longer illness duration. These findings, which were unrelated to OCD symptom severity, complement previous evidence of differences between adult and pediatric OCD patients in brain morphological^3,10,44^ and clinical^45^ correlates.

There is evidence that the human brain’s protracted myelination^46,47^ underpins myelin vulnerability along a continuum from early to late stages of development and disease^48^. Thus, it has been suggested that pediatric OCD could be a neurodevelopmental disorder with potentially differing patterns of myelination occurring throughout life^49^. Indeed, evidence in healthy subjects indicates that the psychiatric trait of compulsivity is linked to reduced myelin growth that emerges only during adolescence (being present only to a minor extent in childhood) as a result of aberrant developmental processes^23^. Alternatively, pediatric OCD might be a developmentally moderated expression of etiologic processes that are shared with the adult clinical phenotype.

A number of limitations of the data analyzed here deserve emphasis. First, although TBSS is a widely used method for voxel-based analysis of WM, addressing issues associated with smoothing and misalignment in DTI group analysis^37^, the technique has some limitations. Indeed, by reducing WM tracts into a skeleton, delineating the center of the tracts and projecting onto it only the highest FA value along the projection, some information might be lost^50^, and potential artifacts, resulting from misregistration, might be produced^51^. Nevertheless, several test–retest and reliability analyses were conducted by the ENIGMA-DTI working group to ensure reproducibility of measures and effects using this TBSS approach^52^. Future investigations combining various imaging modalities in the same meta-analysis and both pre-analyzed and raw data^53^ will potentially offer insights that are not apparent from the TBSS approach. Moreover, a word of caution is needed regarding the interpretation of the neurobiological basis of DTI measures since although FA reflects the myelination, orientational coherence, and microtubular axonal structure of fibers, other in vivo markers not explored in the present study have been shown to be a more direct reflection of myelination status^54,55^. Another potential limitation of the present study may lie in the differences in clinical characteristics between the studied patients, particularly in the average age of onset (which ranged from 4 to 49). Since the latter is often calculated retrospectively, a reliable and unanimous method for establishing this important effect moderator is warranted. Also, we were not able to calculate specific dosages of different medication types and analyze medication effects in terms of drug dosages or total time of treatment and, as such, potential detrimental/normalizing effects of different medications could not be tested. Furthermore, while disorder severity was assessed cross-sectionally at the time of the scan, WM integrity reflects a process that occurred longitudinally over time and this could explain the absence of disorder severity effects. Lastly, it is worth mentioning that while the adult cohort analysis had sufficient power to detect the observed effect size, as the sample size was adequate to detect microstructural differences as small as *d* = 0.15, the null result in the pediatric cohort may be a consequence of the relatively small sample size since the power for potentially detecting even very small differences was low (0.32). Nevertheless, this is the largest pediatric dataset investigated in a DTI study of OCD.

In summary, our results clearly indicate a key role in adult OCD for microstructural alterations in projection and association fibers to posterior brain regions. Our meta-regression results related to duration suggest that microstructural alterations may persist during the course of the illness, although longitudinal data are needed to confirm such trajectories. Future studies to investigate the co-occurrence of abnormal WM microstructure, GM volume and metabolic differences in OCD may shed light on the interactions and trajectories of structural and functional alterations in this condition. In particular, longitudinal designs, and collecting information from patients at their illness onset, combined with multimodal MRI approaches, such as volumetric, DTI, fMRI, and MRS will help provide an understanding of the timing and course of brain changes in OCD, and provide greater insight into the mechanisms involved in various stages of OCD, including the long-term effects of medication.

## Supplementary information

Supplementary material

## References

[1] Micali N (2010). Long-term outcomes of obsessive-compulsive disorder: Follow-up of 142 children and adolescents. Br. J. Psychiatry.

[2] Basser PJ, Pierpaoli C (1996). Microstructural and physiological features of tissues elucidated by quantitative-diffusion-tensor MRI. J. Magn. Reson..

[3] Radua J (2014). Multimodal voxel-based meta-analysis of white matter abnormalities in obsessive-compulsive disorder. Neuropsychopharmacology.

[4] Jenkins LM (2016). Shared white matter alterations across emotional disorders: A voxel-based meta-analysis of fractional anisotropy. NeuroImage Clin..

[5] Koch K, Reeß TJ, Rus OG, Zimmer C, Zaudig M (2014). Diffusion tensor imaging (DTI) studies in patients with obsessive-compulsive disorder (OCD): A review. J. Psychiatr. Res..

[6] Eng GK, Sim K, Chen SHA (2015). Meta-analytic investigations of structural grey matter, executive domain-related functional activations, and white matter diffusivity in obsessive compulsive disorder: an integrative review. Neurosci. Biobehav. Rev..

[7] Melicher T (2015). White matter changes in first episode psychosis and their relation to the size of sample studied: A DTI study. Schizophr. Res..

[8] Thompson PM (2017). ENIGMA and the individual: predicting factors that affect the brain in 35 countries worldwide. Neuroimage.

[9] Boedhoe PSW (2017). Distinct subcortical volume alterations in pediatric and adult OCD: a worldwide meta- and mega-analysis. Am. J. Psychiatry.

[10] Boedhoe PSW (2018). Cortical abnormalities associated with pediatric and adult obsessive-compulsive disorder: findings from the enigma obsessive-compulsive disorder working group. Am. J. Psychiatry.

[11] Piras F, Piras F, Caltagirone C, Spalletta G (2013). Brain circuitries of obsessive compulsive disorder: a systematic review and meta-analysis of diffusion tensor imaging studies. Neurosci. Biobehav Rev..

[12] First, M. B., Spitzer, R. L., Gibbon, M., & Williams, J. B. W. Structured clinical interview for DSM-IV-TR axis I disorders, researchversion, patient edition. (SCID-I/P). (Biometrics Research, New York State Psychiatric Institute, New York, 2002).

[13] Sheehan DV (1998). The Mini-International Neuropsychiatric Interview (M.I.N.I.): the development and validation of a structured diagnostic psychiatric interview for DSM-IV and ICD-10. J. Clin. Psychiatry.

[14] Kaufman, J., & Schweder, A. E. The Schedule for Affective Disorders and Schizophrenia for School - Age Children: Present and Lifetime version (K-SADS-PL) in *The Comprehensive Handbook of Psychological Assessment*. *(CHOPA)**Volume 2: Personality Assessment* Vol. 2 (eds. Hersen, M., Segal, D. M. & Hilsenroth, M. J.) 247–255 (John Wiley & Sons Inc., Hoboken, NJ, 2003).

[15] Goodman, W. K. et al. The Yale-brown Obsessive Compulsive Scale. I. Development, use, and reliability. *Arch. Gen. psychiatry* **46**, 1006–1011, http://www.ncbi.nlm.nih.gov/pubmed/2684084 (1989). 10.1001/archpsyc.1989.01810110048007 2684084

[16] Scahill L (1997). Children’s Yale-Brown Obsessive Compulsive Scale: reliability and validity. J. Am. Acad. Child Adolesc. Psychiatry.

[17] Kelly S (2018). Widespread white matter microstructural differences in schizophrenia across 4322 individuals: results from the ENIGMA Schizophrenia DTI Working Group. Mol. Psychiatry.

[18] van Velzen LS (2020). White matter disturbances in major depressive disorder: a coordinated analysis across 20 international cohorts in the ENIGMA MDD working group. Mol. Psychiatry.

[19] Villalón-Reina JE (2020). Altered white matter microstructure in 22q11.2 deletion syndrome: a multisite diffusion tensor imaging study. Mol. Psychiatry.

[20] Smith SMSSM (2006). Tract-based spatial statistics: voxelwise analysis of multi-subject diffusion data. Neuroimage.

[21] Mori S (2008). Stereotaxic white matter atlas based on diffusion tensor imaging in an ICBM template. Neuroimage.

[22] Nave K-A, Ehrenreich H (2014). Myelination and oligodendrocyte functions in psychiatric diseases. JAMA Psychiatry.

[23] Ziegler G (2019). Compulsivity and impulsivity traits linked to attenuated developmental frontostriatal myelination trajectories. Nat. Neurosci..

[24] Kessler RC (2007). Lifetime prevalence and age-of-onset distributions of mental disorders in the World Health Organization’s World Mental Health Survey Initiative. World Psychiatry.

[25] Paus T, Keshavan M, Giedd JN (2008). Why do many psychiatric disorders emerge during adolescence?. Nat. Rev. Neurosci..

[26] Song S (2003). Diffusion tensor imaging detects and differentiates axon and myelin degeneration in mouse optic nerve after retinal ischemia. Neuroimage.

[27] Rosso IM (2014). Brain white matter integrity and association with age at onset in pediatric obsessive-compulsive disorder. Biol. Mood Anxiety Disord..

[28] Alexander AL (2011). Characterization of cerebral white matter properties using quantitative magnetic resonance imaging stains. Brain Connect..

[29] Maia TV, Cooney RE, Peterson BS (2008). The neural bases of obsessive - compulsive disorder in children and adults. Dev. Psychopathol..

[30] Fields RD (2008). White matter in learning, cognition and psychiatric disorders. Trends Neurosci..

[31] Fields RD (2015). A new mechanism of nervous system plasticity: activity-dependent myelination. Nat. Rev. Neurosci..

[32] Kim, S. J., Lewis, M. & Veenstra-VanderWeele J. The Developmental Neurobiology of Repetitive Behavior. In: *Neural Circuit Development and Function in the Heathy and Diseased Brain* (eds Rubenstein, J. L. & Rakic, P.) (Academic Press, 2013).

[33] Benedetti F (2013). Widespread changes of white matter microstructure in obsessive-compulsive disorder: effect of drug status. Eur. Neuropsychopharmacol..

[34] Haroutunian V (2014). Myelination, oligodendrocytes, and serious mental illness. Glia.

[35] Káradóttir R, Attwell D (2007). Neurotransmitter receptors in the life and death of oligodendrocytes. Neuroscience.

[36] Bollettini I (2018). White matter alterations associate with onset symptom dimension in obsessive–compulsive disorder. Psychiatry Clin. Neurosci..

[37] Gan J (2017). Abnormal white matter structural connectivity in adults with obsessive-compulsive disorder. Transl. Psychiatry.

[38] Calzà, J. et al. Altered cortico-striatal functional connectivity during resting state in obsessive-compulsive disorder. *Front. Psychiatry* **10**, 319, https://www.frontiersin.org/article/10.3389/fpsyt.2019.00319/full (2019). 10.3389/fpsyt.2019.00319 PMC6524661 31133898

[39] Garibotto V (2010). Disorganization of anatomical connectivity in obsessive compulsive disorder: A multi-parameter diffusion tensor imaging study in a subpopulation of patients. Neurobiol. Dis..

[40] Kim SG, Jung WH, Kim SN, Jang JH, Kwon JS (2015). Alterations of gray and white matter networks in patients with obsessive-compulsive disorder: Amultimodal fusion analysis of structural MRI and DTI using mCCA + jICA. PLoS ONE.

[41] Hou JM (2014). Resting-state functional connectivity abnormalities in patients with obsessive compulsive disorder and their healthy first-degree relatives. J. Psychiatry Neurosci..

[42] Moreira PS (2017). The neural correlates of obsessive-compulsive disorder: a multimodal perspective. Transl. Psychiatry.

[43] Menzies L (2008). White matter abnormalities in patients with obsessive-compulsive disorder and their first-degree relatives. Am. J. Psychiatry.

[44] Pujol, J. et al. Mapping structural brain alterations in obsessive-compulsive disorder. *Arch. Gen. Psychiatry* **61**, 720–730, http://archpsyc.jamanetwork.com/article.aspx?doi=10.1001/archpsyc.61.7.720 (2004). 10.1001/archpsyc.61.7.720 15237084

[45] Geller DA (2001). Developmental aspects of obsessive compulsive disorder: findings in children, adolescents, and adults. J. Nerv. Ment. Dis..

[46] de Graaf-Peters VB, Hadders-Algra M (2006). Ontogeny of the human central nervous system: What is happening when?. Early Hum. Dev..

[47] Bartzokis GB (2005). Brain myelination in prevalent neuropsychiatric developmental disorders: Primary and comorbid addiction. Adolesc. Psychiatry.

[48] Sherin, J. E. & Bartzokis, G. Human Brain Myelination Trajectories Across the Life Span: Implications for CNS Function and Dysfunction. In: *Handbook of the Biology of Aging* (eds Masoro, E. J. & Austad, S. N.) 336–446 (Academic Press, 2011).

[49] Gruner, P. et al. White matter abnormalities in pediatric obsessive-compulsive disorder. *Neuropsychopharmacology* **37**, 2730–2739, http://www.pubmedcentral.nih.gov/articlerender.fcgi?artid=3473339&tool=pmcentrez&rendertype=abstract (2012). 10.1038/npp.2012.138 PMC3473339 22871914

[50] Zalesky A (2011). Moderating registration misalignment in voxelwise comparisons of DTI data: A performance evaluation of skeleton projection. Magn. Reson. Imaging.

[51] Schwarz CG (2014). Improved DTI registration allows voxel-based analysis that outperforms Tract-Based Spatial Statistics. Neuroimage.

[52] Acheson A (2017). Reproducibility of tract-based white matter microstructural measures using the ENIGMA-DTI protocol. Brain Behav..

[53] Radua J, Mataix-Cols D (2012). Meta-analytic methods for neuroimaging data explained. Biol. Mood Anxiety Disord..

[54] Schmierer K, Scaravilli F, Altmann DR, Barker GJ, Miller DH (2004). Magnetization transfer ratio and myelin in postmortem multiple sclerosis brain. Ann. Neurol..

[55] Turati L (2015). In vivo quantitative magnetization transfer imaging correlates with histology during de- and remyelination in cuprizone-treated mice. NMR Biomed..

