# Supplementary material for: White matter microstructure and its relation to clinical features of obsessive–compulsive disorder: findings from the ENIGMA OCD Working Group
Source: Transl Psychiatry. 2021 Mar 17;11:173. doi: 10.1038/s41398-021-01276-z (PMC7969744; doi:10.1038/s41398-021-01276-z)
Supplement: Supplementary file 1 — Supplementary material [file 41398_2021_1276_MOESM1_ESM.docx]

Supplementary Table 1.

Medication class

Adult sample

| **Site** | **OCD/HC (N)** | **Medicated (%)** | **Antidepressants (%)** | **Antipsychotics (%)** | **Antidepressants + Antipsychotics** |
| --- | --- | --- | --- | --- | --- |
| Amsterdam | 38/34 | 0 | - | - | - |
| Bangelore | 158/131 | 39,9 | NA | NA | NA |
| Capetown | 22/23 | 40,9 | 31,8 | 0 | 9,1 |
| Kyoto | 35/41 | 0 | - | - | - |
| Milan | 63/65 | 60,3 | 49,2 | 3,2 | 7,9 |
| Mount Sinai | 16/18 | 81,3 | NA | NA | NA |
| Munich | 73/60 | 60,3 | NA | NA | NA |
| Rome | 77/111 | 94,8 | 42,8 | 22 | 30 |
| Sao Paulo | 37/30 | 43,2 | NA | NA | NA |
| Shangai | 83/45 | 0 | - | - | - |
| Seoul | 98/87 | 13,3 | NA | NA | NA |

Pediatric sample

| **Site** | **OCD/HC (N)** | **Medicated (%)** | **Antidepressants (%)** | **Antipsychotics (%)** | **Antidepressants + Antipsychotics** |
| --- | --- | --- | --- | --- | --- |
| Bangelore | 13/13 | 85 | NA | NA | NA |
| Barcelona | 52/27 | 78,8 | 78,8 | 0 | 0 |
| British Columbia | 13/16 | 86,7 | NA | NA | NA |
| Calgary | 19/18 | 0 | - | - | - |
| Chiba | 20/6 | 40 | 35 | 5 | 0 |
| Oxford | 13/18 | 63,6 | 59 | 2,3 | 2,3 |
| Yale | 22/23 | 52,2 | NA | NA | NA |
| Zurich | 22/23 | 57,1 | 42,85 | 7,13 | 7,13 |

Supplementary Table 2.

Sensitivity analyses through leave-one-out procedure. Sites which removal determined a loss of significance are in red.

| **GCC** |  |  |  |  |  |  |  |  |
| --- | --- | --- | --- | --- | --- | --- | --- | --- |
| Site | Tract | Point | SE | Variance | Lower limit | Upper limit | Z-Value | p-Value |
| Amsterdam | GCC | -0,1922 | 0,0895 | 0,0080 | -0,3677 | -0,0167 | -2,1466 | 0,0318 |
| Bangelore | GCC | -0,2136 | 0,0834 | 0,0070 | -0,3771 | -0,0501 | -2,5601 | 0,0105 |
| Capetown | GCC | -0,1706 | 0,0902 | 0,0081 | -0,3473 | 0,0062 | -1,8911 | 0,0586 |
| Kyoto | GCC | -0,1887 | 0,0907 | 0,0082 | -0,3665 | -0,0110 | -2,0807 | 0,0375 |
| Milan | GCC | -0,1099 | 0,0742 | 0,0055 | -0,2554 | 0,0355 | -1,4810 | 0,1386 |
| Mount Sinai | GCC | -0,1530 | 0,0868 | 0,0075 | -0,3231 | 0,0171 | -1,7628 | 0,0779 |
| Munich | GCC | -0,1889 | 0,0933 | 0,0087 | -0,3718 | -0,0060 | -2,0246 | 0,0429 |
| Rome | GCC | -0,1409 | 0,0889 | 0,0079 | -0,3152 | 0,0334 | -1,5841 | 0,1132 |
| Sao Paulo | GCC | -0,1381 | 0,0843 | 0,0071 | -0,3034 | 0,0272 | -1,6380 | 0,1014 |
| Seoul | GCC | -0,1863 | 0,0957 | 0,0092 | -0,3738 | 0,0013 | -1,9469 | 0,0515 |
| Shangai | GCC | -0,1846 | 0,0934 | 0,0087 | -0,3676 | -0,0016 | -1,9771 | 0,0480 |
| Random |  | -0,1696 | 0,0845 | 0,0071 | -0,3352 | -0,0041 | -2,0085 | 0,0446 |
| **PCR** |  |  |  |  |  |  |  |  |
| Site | Tract | Point | SE | Variance | Lower limit | Upper limit | Z-Value | p-Value |
| Amsterdam | PCR | -0,1771 | 0,0669 | 0,0045 | -0,3083 | -0,0459 | -2,6454 | 0,0082 |
| Bangelore | PCR | -0,1739 | 0,0762 | 0,0058 | -0,3233 | -0,0245 | -2,2813 | 0,0225 |
| Capetown | PCR | -0,1534 | 0,0706 | 0,0050 | -0,2918 | -0,0151 | -2,1741 | 0,0297 |
| Kyoto | PCR | -0,1785 | 0,0664 | 0,0044 | -0,3087 | -0,0483 | -2,6877 | 0,0072 |
| Milan | PCR | -0,1216 | 0,0626 | 0,0039 | -0,2443 | 0,0011 | -1,9426 | 0,0521 |
| Mount Sinai | PCR | -0,1558 | 0,0703 | 0,0049 | -0,2936 | -0,0180 | -2,2159 | 0,0267 |
| Munich | PCR | -0,1467 | 0,0729 | 0,0053 | -0,2896 | -0,0037 | -2,0110 | 0,0443 |
| Rome | PCR | -0,1284 | 0,0690 | 0,0048 | -0,2636 | 0,0069 | -1,8601 | 0,0629 |
| Sao Paulo | PCR | -0,1351 | 0,0652 | 0,0043 | -0,2628 | -0,0073 | -2,0715 | 0,0383 |
| Seoul | PCR | -0,1916 | 0,0659 | 0,0043 | -0,3208 | -0,0624 | -2,9067 | 0,0037 |
| Shangai | PCR | -0,1645 | 0,0734 | 0,0054 | -0,3083 | -0,0207 | -2,2424 | 0,0249 |
| Random |  | -0,1570 | 0,0660 | 0,0043 | -0,2863 | -0,0277 | -2,3803 | 0,0173 |
| **PTR** |  |  |  |  |  |  |  |  |
| Site | Tract | Point | SE | Variance | Lower limit | Upper limit | Z-Value | p-Value |
| Amsterdam | PTR | -0,2469 | 0,0583 | 0,0034 | -0,3612 | -0,1327 | -4,2350 | 0,0000 |
| Bangelore | PTR | -0,2234 | 0,0617 | 0,0038 | -0,3443 | -0,1025 | -3,6216 | 0,0003 |
| Capetown | PTR | -0,2630 | 0,0621 | 0,0039 | -0,3847 | -0,1412 | -4,2336 | 0,0000 |
| Kyoto | PTR | -0,2658 | 0,0629 | 0,0040 | -0,3890 | -0,1425 | -4,2267 | 0,0000 |
| Milan | PTR | -0,2447 | 0,0612 | 0,0037 | -0,3646 | -0,1247 | -3,9984 | 0,0001 |
| Mount Sinai | PTR | -0,2575 | 0,0613 | 0,0038 | -0,3776 | -0,1374 | -4,2021 | 0,0000 |
| Munich | PTR | -0,2686 | 0,0642 | 0,0041 | -0,3945 | -0,1428 | -4,1827 | 0,0000 |
| Rome | PTR | -0,2589 | 0,0660 | 0,0044 | -0,3883 | -0,1294 | -3,9199 | 0,0001 |
| Sao Paulo | PTR | -0,2497 | 0,0599 | 0,0036 | -0,3671 | -0,1323 | -4,1698 | 0,0000 |
| Seoul | PTR | -0,3067 | 0,0590 | 0,0035 | -0,4224 | -0,1910 | -5,1955 | 0,0000 |
| Shangai | PTR | -0,2921 | 0,0574 | 0,0033 | -0,4046 | -0,1796 | -5,0894 | 0,0000 |
| Random |  | -0,2619 | 0,0573 | 0,0033 | -0,3742 | -0,1495 | -4,5689 | 0,0000 |
| **SS** |  |  |  |  |  |  |  |  |
| Site | Tract | Point | SE | Variance | Lower limit | Upper limit | Z-Value | p-Value |
| Amsterdam | SS | -0,208 | 0,071 | 0,005 | -0,347 | -0,068 | -2,920 | 0,003 |
| Bangelore | SS | -0,219 | 0,076 | 0,006 | -0,368 | -0,069 | -2,865 | 0,004 |
| Capetown | SS | -0,201 | 0,069 | 0,005 | -0,337 | -0,066 | -2,912 | 0,004 |
| Kyoto | SS | -0,237 | 0,057 | 0,003 | -0,349 | -0,125 | -4,163 | 0,000 |
| Milan | SS | -0,165 | 0,057 | 0,003 | -0,278 | -0,053 | -2,881 | 0,004 |
| Mount Sinai | SS | -0,209 | 0,070 | 0,005 | -0,345 | -0,072 | -3,002 | 0,003 |
| Munich | SS | -0,212 | 0,073 | 0,005 | -0,355 | -0,069 | -2,898 | 0,004 |
| Rome | SS | -0,196 | 0,073 | 0,005 | -0,340 | -0,053 | -2,675 | 0,007 |
| Sao Paulo | SS | -0,203 | 0,070 | 0,005 | -0,341 | -0,065 | -2,880 | 0,004 |
| Seoul | SS | -0,204 | 0,075 | 0,006 | -0,350 | -0,058 | -2,737 | 0,006 |
| Shangai | SS | -0,240 | 0,063 | 0,004 | -0,363 | -0,117 | -3,827 | 0,000 |
| Random |  | -0,209 | 0,065 | 0,004 | -0,337 | -0,081 | -3,210 | 0,001 |
| **UNC** |  |  |  |  |  |  |  |  |
| Site | Tract | Point | SE | Variance | Lower limit | Upper limit | Z-Value | p-Value |
| Amsterdam | UNC | -0,1936 | 0,0779 | 0,0061 | -0,3462 | -0,0410 | -2,4862 | 0,0129 |
| Bangelore | UNC | -0,1992 | 0,0840 | 0,0071 | -0,3638 | -0,0346 | -2,3717 | 0,0177 |
| Capetown | UNC | -0,1824 | 0,0774 | 0,0060 | -0,3342 | -0,0307 | -2,3558 | 0,0185 |
| Kyoto | UNC | -0,1706 | 0,0776 | 0,0060 | -0,3226 | -0,0185 | -2,1988 | 0,0679 |
| Milan | UNC | -0,2034 | 0,0779 | 0,0061 | -0,3560 | -0,0508 | -2,6128 | 0,0090 |
| Mount Sinai | UNC | -0,1520 | 0,0665 | 0,0044 | -0,2823 | -0,0216 | -2,2848 | 0,0223 |
| Munich | UNC | -0,1784 | 0,0807 | 0,0065 | -0,3366 | -0,0202 | -2,2101 | 0,0271 |
| Rome | UNC | -0,1347 | 0,0702 | 0,0049 | -0,2722 | 0,0028 | -1,9197 | 0,0549 |
| Sao Paulo | UNC | -0,1508 | 0,0705 | 0,0050 | -0,2890 | -0,0126 | -2,1383 | 0,0325 |
| Seoul | UNC | -0,2122 | 0,0752 | 0,0057 | -0,3597 | -0,0647 | -2,8203 | 0,0048 |
| Shangai | UNC | -0,2059 | 0,0759 | 0,0058 | -0,3548 | -0,0571 | -2,7115 | 0,0067 |
| Random |  | -0,1799 | 0,0723 | 0,0052 | -0,3216 | -0,0382 | -2,4885 | 0,0128 |
